# Mendelian randomization study shows a causal effect of asthma on epilepsy risk

**DOI:** 10.3389/fimmu.2023.1071580

**Published:** 2023-02-13

**Authors:** Peng Tang, Xingzhi Guo, Li Chong, Rui Li

**Affiliations:** ^1^ Department of Geriatric Neurology, Shaanxi Provincial People’s Hospital, Xi’an, Shaanxi, China; ^2^ Shaanxi Provincial Clinical Research Center for Geriatric Medicine, Xi’an, Shaanxi, China; ^3^ Institute of Medical Research, Northwestern Polytechnical University, Xi’an, Shaanxi, China

**Keywords:** asthma, epilepsy, genome-wide association study, Mendelian randomization, inverse-variance weighted

## Abstract

**Objective:**

The relationship between asthma and epilepsy in observational studies is controversial. The purpose of this Mendelian randomization (MR) study is to investigate whether asthma causally contributes to epilepsy susceptibility.

**Methods:**

Independent genetic variants strongly (P<5E-08) associated with asthma were from a recent meta-analysis of genome-wide association studies on 408,442 participants. Two independent summary statistics of epilepsy obtained from the International League Against Epilepsy Consortium (ILAEC, Ncases=15,212, and Ncontrols=29,677) and FinnGen Consortium (Ncases=6,260 and Ncontrols=176,107) were used in the discovery and replication stage, respectively. Several sensitivity analyses and heterogeneity analyses were further conducted to assess the stability of the estimates.

**Results:**

Using the inverse-variance weighted approach, genetic predisposition to asthma was associated with an elevated risk of epilepsy in the discovery stage (ILAEC: odds ratio [OR]=1.112, 95% confidence intervals [CI]= 1.023-1.209, *P* = 0.012), but not verified in the replication stage (FinnGen: OR=1.021, 95%CI= 0.896–1.163, *P* =0.753). However, a further meta-analysis of both ILAEC and FinnGen showed a similar result (OR=1.085, 95% CI: 1.012-1.164, *P* = 0.022). There were no causal associations between the age onset of asthma and epilepsy. Sensitivity analyses yielded consistent causal estimates.

**Conclusion:**

The present MR study suggests that asthma is associated with an increased risk of epilepsy independent of the age onset of asthma. Further studies are warranted to explain the underlying mechanisms of this association.

## Introduction

Asthma is one of the most common chronic respiratory disorders (1), affecting affects over 300 million people worldwide and bringing a huge economic and social burden (2). Accumulating evidence has shown that inflammation might be involved in the pathogenesis of asthma (3) and individuals with brain inflammation have a likelihood of being predisposed to epileptogenesis (4, 5). These findings have drawn much attention to exploring the association of asthma with epilepsy. Indeed, two previously published population-based studies of adults revealed that patients with epilepsy were often accompanied by physical comorbidities such as asthma (6, 7). In addition, numerous case-control studies have announced that the prevalence of asthma was related to higher odds of epilepsy either in children (8) or in adults (9). These data suggest that asthma might be associated with high susceptibility to epilepsy. However, data from other case-control studies have displayed discordant findings, with a retrospective study among children suggesting that idiopathic epilepsy is not etiologically connected with asthma (10). Furthermore, observational studies cannot prove the causal inference due to their sensitivities to residual confounding and reverse causality.

Mendelian randomization (MR), using genetic connections to inquire about the causal impact of a risk factor on an outcome (11), is an effective method for gaging causal inference. This approach can not only limit reverse causality but also greatly reduce the likelihood of residual confounding (12). Based on the inconsistent findings of the aforementioned retrospective cohort studies, we undertook a 2-sample MR approach to assess whether asthma causally contributed to an increased risk of epilepsy.

## Methods

### Study design and data source

**Figure 1:**
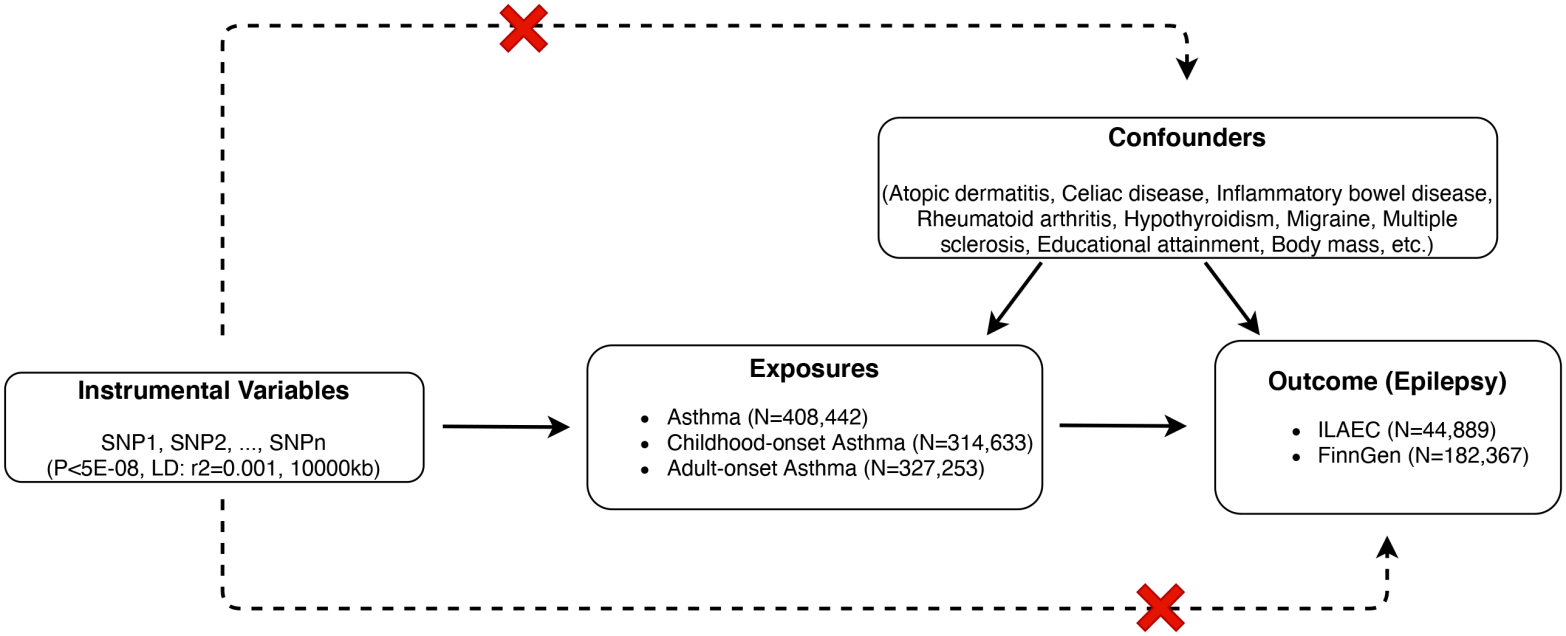
Three corresponding principal assumptions in this 2-sample Mendelian randomization study. Red stars mean that genetic variants are not associated with confounding factors and the outcome.

Independent single nucleotide polymorphisms (SNPs) from genome-wide association studies (GWAS) were selected as instrumental variables (IV). This MR study aimed to satisfy the three primary assumptions described in detail in **Figure 1**. Assumption 1 (Relevance), SNPs significantly (P<5E-08) associated with asthma. Assumption 2 (Independence), SNPs not associated with confounding factors that correlated with both asthma and epilepsy, including atopic dermatitis (13), celiac disease (14), inflammatory bowel disease (15), rheumatoid arthritis (16), hypothyroidism (17), migraine (18), multiple sclerosis (19), educational attainment (20), and body mass (21). Assumption 3 (Exclusivity), SNPs affected epilepsy susceptibility directly through asthma and are not associated with epilepsy (P>1E-05).

The summary statistics of asthma were from the latest large-scale GWAS meta-analysis of 408,442 Europeans from the UK Biobank (22). For childhood-onset and adult-onset asthma (23), there were 314,633 and 327,253 participants of European descent from the UK Biobank, respectively. For epilepsy, two independent summary statistics of epilepsy from the International League Against Epilepsy Consortium (ILAEC) and the FinnGen Consortium were included in this MR study. The summary statistics from the ILAEC contained 15,212 cases and 29,677 normal controls (24), and a total of 6,260 epilepsy cases and 176,107 normal controls of European descent were obtained from the FinnGen Consortium (25). Since samples from the ILAEC had a higher proportion of cases (33.9%) than those from the FinnGen Consortium (3.5%), we used the datasets of ILAEC and FinnGen Consortium in the discovery stage and replication stage, respectively. Epilepsy was diagnosed by epilepsy specialists based on electroencephalography, magnetic resonance imaging, and clinical history. **Table 1** includes a detailed summary of the study including source publications (**Table 1**).

**Table 1:** Summary of the genome-wide association studies included in this Mendelian randomization study.

Phenotype	Author	Year	Sample size (N)	SNP(N)	PMID	URL (Data Download)
Asthma	Valette et al.	2021	408,442	34,551,291	34103634	https://www.ebi.ac.uk/gwas/downloads/summary-statistics
Asthma (adult-onset)	Ferreira et al.	2019	327,253	8,949,308	30929738	https://www.ebi.ac.uk/gwas/downloads/summary-statistics
Asthma (childhood-onset)	Ferreira et al.	2019	314,633	8,984,776	30929738	https://www.ebi.ac.uk/gwas/downloads/summary-statistics
Epilepsy						
ILAEC	Abou-Khalil et al.	2018	44,889	4,880,492	30531953	https://gwas.mrcieu.ac.uk/files/ieu-b-8/ieu-b-8.vcf.gz
FinnGen	FinnGen project	2021	182,367	16,380,349	–	https://finngen.gitbook.io/documentation/data-download

SNP, single nucleotide polymorphism; N, number.

### Instruments selection

Those SNPs passing the genome-wide significance threshold (P < 5E–08) were selected as IVs, which were clumped according to the linkage disequilibrium structure (1000 Genomes Project of European, r^2^<0.01 within 10000 kb). In addition, SNPs associated with epilepsy with a P value lower than 1E–05 were excluded from the IV before MR analysis. Meanwhile, IVs associated with the confounders described above were also removed from the MR analysis. SNPs absent from the epilepsy GWAS datasets will be replaced with overlapping proxy SNPs (r^2 =^ 0.8). To strengthen the robustness of the estimates, SNPs with a minor allele frequency of less than 0.3 were also removed. All harmonized SNPs for each exposure-outcome pair were archived (**Supplementary Data Sheet**).

### Mendelian randomization analysis

The TwoSampleMR package (version 0.5.6) was applied in the present Mendelian randomization analysis (26). The inverse-variance weighted (IVW) method was used as the default method to calculate causal estimates between asthma and epilepsy. Meanwhile, we also employed weighted median, MR–Egger regression, weighted mode, simple median, maximum likelihood, and MR-Pleiotropy RESidual Sum and Outlier (MR-PRESSO) as sensitivity analyses to validate the estimates (27). MR-PRESSO test could identify horizontal pleiotropic outliers and evaluate the potential pleiotropic effects of the genetic variants selected as IV. MR–Egger intercept test was also applied to measure the horizontal pleiotropy. In addition, F-statistics were also calculated to assess the instrumental strength as previously described (28), and F values of more than 10 were found to avoid bias from weak instruments.

A meta-analysis based on ILAEC and FinnGen was also conducted to calculate the overall causal estimates using the meta package (version 5.2.0). A fixed-effect model was applied to combine the estimates if there was obvious heterogeneity (P>0.05 or I^2^<50%), otherwise, a random-effect model was employed (29). There is yet a lack of consensus regarding the best strategy for multiple test correction (30, 31), where multiple testing for different outcomes might increase the risk of Type I error, while adjustment for multiple comparisons could increase the risk of type II errors. To balance the type I and type II errors, we followed the strategy reported previously by Ronald J. Feise *via* conducting independent Bonferroni correction for each outcome assessed (30). Since two independent GWAS datasets for epilepsy were included in this study, a P-value < 0.025 after Bonferroni correction (0.05/2) was considered statistically significant. Meanwhile, a P-value < 0.05 was considered suggestive of a causal association. All statistical analyses were performed in R software (version 4.1.3), and the meta package (version 5.2.0) and forestploter package (version 0.1.5) was employed in drawing forest plots.

## Results

**Figure 2:**
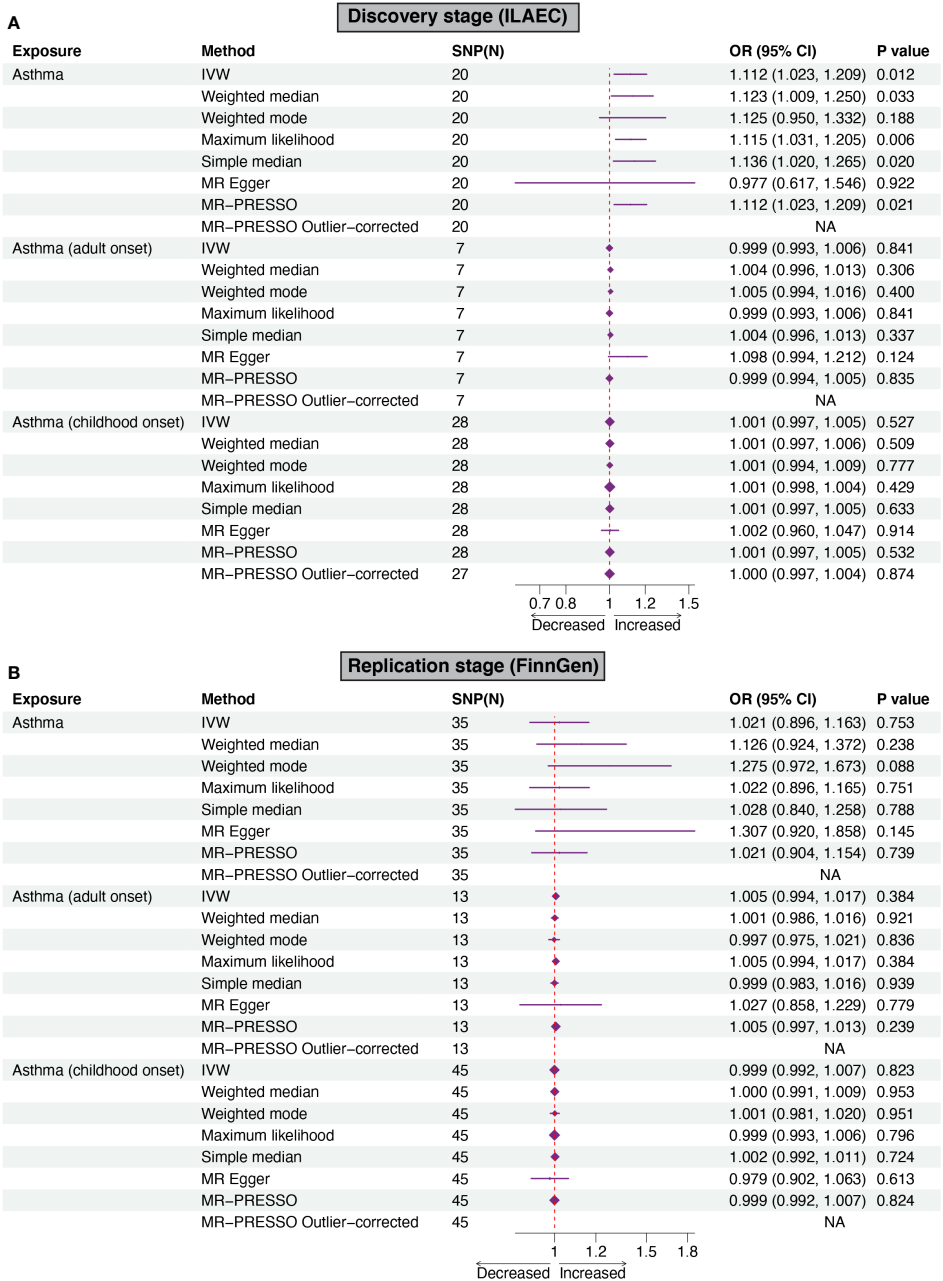
Forest plots of Mendelian randomization analyses show the causal effects of asthma on epilepsy. Six different methods, including IVW, weighted mode, weighted median, MR-Egger regression, MR-PRESSO, simple median, and maximum likelihood were used to evaluate the causal effect of asthma on epilepsy. **(A, B)** showed the causal effect of asthma on epilepsy in the discovery stage and replication stage, respectively. IVW, inverse variance weighed MR-PRESSO, MR-Pleiotropy RESidual Sum, and Outlier.

Using the IVW method, genetically predicted asthma was associated with an increased risk of epilepsy in the discovery stage (ILAEC: OR = 1.112, 95% CI: 1.023-1.209, *P* = 0.012). Directional consistent results were obtained in sensitivity analyses using simple median, weighted median, maximum likelihood, and MR-PRESSO approaches (**Figure 2A**). In the replication stage, estimates of the FinnGen dataset showed the same trend direction as the results of ILAEC (**Figure 2B**). No obvious causal effects of childhood-onset asthma and adult-onset asthma on epilepsy were found in both the discovery stage and replication stage (**Figure 2**). There was no obvious pleiotropy observed in the MR-Egger intercept test, but potential pleiotropy of childhood-onset asthma on epilepsy (P=0.037) in the discovery stage was observed in the MR-PRESSO test (**Table 2**). Cochran-Q test also showed heterogeneity in evaluating the causal association between childhood-onset asthma and epilepsy in the discovery stage (**Table 2**). The corrected estimate after removing the outlier (rs1893380) identified by the MR-PRESSO test showed a similar result, suggesting good stability. All the F-statistic values were larger than 10 across the MR study, indicating good instrumental strength.

**Table 2:** Heterogeneity and power analysis of asthma on epilepsy.

Exposure\Outcome	Method	ILAEC (epilepsy)				FinnGen Consortium (epilepsy)			
		MR-Egger intercept (P)	MR_PRESSO (P)	F-statistic	Cochran-Q (P)	MR-Egger intercept (P)	MR_PRESSO (P)	F-statistic	Cochran-Q (P)
**Asthma**	IVW	0.01 (0.580)	24.50 (0.300)	44.35	22.45 (0.262) 22.06 (0.229)	-0.02 (0.148)	33.87 (0.580)	326.03	29.86 (0.671) 27.67 (0.730)
	MR-Egger								
**Asthma** **(adult-onset)**	IVW	-0.09 (0.121)	7.09 (0.534)	20.64	5.08 (0.533) 1.61 (0.900)	-0.02 (0.821)	6.99 (0.914)	119.51	5.92 (0.920) 5.87 (0.882)
	MR-Egger								
**Asthma** **(childhood-onset)**	IVW	-0.001 (0.957)	45.19 (0.037)	35.59	42.23 (0.031) 42.23 (0.023)	0.02 (0.625)	61.90 (0.065)	330.98	58.87 (0.066) 58.54 (0.057)
	MR-Egger								

MR, Mendelian randomization; ILAEC, International League Against Epilepsy Consortium; IVW, inverse-variance weighted; MR_PRESSO, Mendelian Randomization Pleiotropy RESidual Sum and Outlier; P, P value.

**Figure 3:**
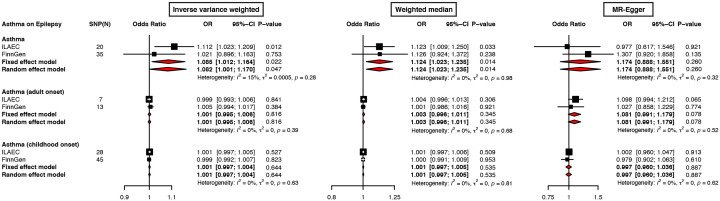
Forest plots of meta-analysis on ILAEC and FinnGen epilepsy GWAS datasets show the causal effects of asthma on epilepsy. The inverse variance weighted, weighted median, and MR-Egger regression were used to evaluate the causal effects of asthma on epilepsy.

A further meta-analysis of ILAEC and FinnGen also showed a causal effect of asthma on epilepsy (OR = 1.085, 95% CI: 1.012-1.164, *P* = 0.022), which was validated in a sensitivity analysis using other approaches (**Figure 3**; **Supplementary Figure S1**). The meta-analysis results from both the fixed-effect model and the random-effect were largely consistent across different statistical methods (**Figure 3**, **Supplementary Figure S1**).

## Discussion

In this study, we took advantage of the 2-sample MR method to analyze the causal relationship between asthma and epilepsy. The main results consistently suggested that asthma was associated with a higher risk of epilepsy. Furthermore, several sensitivity analyses were used based on their different underlying assumptions and similar results were observed, which further strengthened the credibility of the results.

Previous reports have investigated the relationship between asthma and epilepsy, but the results were inconsistent. A population-based study found that most adult patients with epilepsy presently have symptomatic asthma (6). Meanwhile, a U.S. National Health Interview Survey found that adult patients with epilepsy were more often to record physical comorbidities like asthma (7). Previous studies among US children aged 0-17 years reported that the lifetime prevalence of asthma was related to a higher risk of epilepsy (2.30 [1.50-3.52]) (8). Similarly, a recent cohort study including 150,827 asthma patients showed that the asthma patients had an increased risk of epilepsy than health controls (hazard ratio=1.39) (9). All these findings indicated that asthma was associated with the risk of epilepsy, which was consistent with the results of our MR study based on data from the ILAEC and FinnGen Consortium. Although an early study among children suggested that there was no etiological relationship between asthma and epilepsy, the result may be attributed to small samples (10).

The underlying mechanism mediating the association between asthma and epilepsy remains largely unknown. The potential reasons connecting asthma and epilepsy are anoxia and hypocapnia owing to repeated asthma attacks. In addition, chronic inflammation is a common pathological feature shared by asthma and epilepsy (3, 32, 33). Previous studies demonstrated that circulating cytokines might penetrate through the blood-brain barrier and then result in chronic neuroinflammation and neuronal damage, eventually increasing the susceptibility to epileptogenesis (4, 5, 34). Moreover, emerging evidence shows that the respiratory system has a tight relationship with the central nervous system, which goes beyond the classically known connections such as blood supply and oxygen saturation. Studies showed that respiratory system diseases such as asthma (35) and chronic obstructive pulmonary disease (36) might increase the risk of stroke, which was a risk factor for epilepsy. In addition, clinical data suggested that chronic obstructive pulmonary disease was associated with an increased risk for the development of seizures in patients with stroke (37). Although oxygen desaturation may be one of the risk factors for epilepsy in asthma patients (38), further work is needed to explore the exact mechanisms by which asthma causes an increased risk of epilepsy.

Asthma can be divided into childhood-onset asthma and adult-onset asthma based on the age of onset. Childhood-onset asthma may be related to genetic factors (39, 40), perinatal factors (41), or respiratory infections (42), while adult-onset asthma may be related to environmental and occupational factors such as obesity and smoking (43). Even though the mechanisms contributing to childhood-onset and adult-onset asthma might be different, our MR study found no causal associations between the age onset of asthma and epilepsy. These data suggested that asthma causally increased the risk of epilepsy independent of the age onset of asthma. The potential reason for these unexpected results might be due to the small sample size of childhood-onset and adult-onset asthma, which might lead to lower statistical power. It is worth noting that the proportion of cases with asthma was 13.8%, while in childhood-onset asthmatic and adult-onset asthma were 4.4% and 8.1%, respectively. In addition, although the causal relationship was not significant for childhood-onset asthma and adult-onset asthma on epilepsy, most of the OR values were larger than 1, suggesting a potential risk effect of childhood-onset asthma and adult-onset asthma on epilepsy.

This study has some limitations: first, the nonlinear connection between asthma and the risk of epilepsy cannot be eliminated due to the linear effect assumption in MR analysis. Second, although no evidence of pleiotropy was detected in the MR-Egger intercept test, potential pleiotropy was observed between childhood-onset asthma and epilepsy (P=0.037) in the MR-PRESSO test. Third, there was obvious heterogeneity between childhood-onset asthma and epilepsy from ILAEC datasets, which might be due to the mixed population of ILAEC (531 and 147 individuals of Asian and African descent, respectively). Fourth, our study is mainly based on Europeans, thus generalization of the findings to other ethnic groups needs to be cautious. Fifth, to better fulfill the independence assumption for the MR study, we used a relatively stringent way to exclude the SNPs associated with potential confounders of epilepsy from the IVs, which might weaken the statistical power of the MR study. Sixth, due to individual data not being publically available, we were unable to properly account for the potential sample overlap between the GWAS datasets of asthma and epilepsy, which might lead to bias in the overall estimates. Finally, there are other possible unmeasured and residual confounding factors like many other epidemiological studies, which might drive the bias of the overall estimates. For example, as asthma was caused due to an overactive immune response (44), many instrumental variables for asthma were associated with peripheral blood cells (**Supplementary Data Sheets**, **7**). Although previous studies suggested that inflammatory factors were also implicated in epilepsy (33), however, asthma, characterized by chronic inflammation and bronchial hyperresponsiveness, is a disease strongly related to the inflammatory response (45). If all instrumental variables related to peripheral blood cells were excluded, the number of instrumental variables would be dramatically reduced. Thus, like other previously published MR studies on asthma (46, 47), the SNPs related to peripheral blood cells were not removed from the instrumental variables in our MR study, which could not rule out the potential influence of inflammatory factors on the causal relationship between asthma and epilepsy.

In conclusion, the present MR study suggests that asthma is associated with an increased risk of epilepsy independent of the age onset of asthma. Further studies are warranted to investigate the potential mechanism mediating the causal effect of asthma on epilepsy.

## Data availability statement

The original contributions presented in the study are included in the article/**Supplementary Material**. Further inquiries can be directed to the corresponding authors.

## Author contributions

PT, XG, and RL conceived and designed the project. PT, XG, and LC collected and analyzed the data. XG and PT drafted the manuscript. RL revised the manuscript. All authors approved the final version of the manuscript.

## Supplementary material

Supplementary Data Sheet Summary of harmonized instrumental variables used in this MR study.
