# Supplementary material for: Mendelian randomization study shows a causal effect of asthma on epilepsy risk
Source: Front Immunol. 2023 Feb 13;14:1071580. doi: 10.3389/fimmu.2023.1071580 (PMC9969112; doi:10.3389/fimmu.2023.1071580)
Supplement: Supplementary file 2 [file DataSheet_2.docx]

**
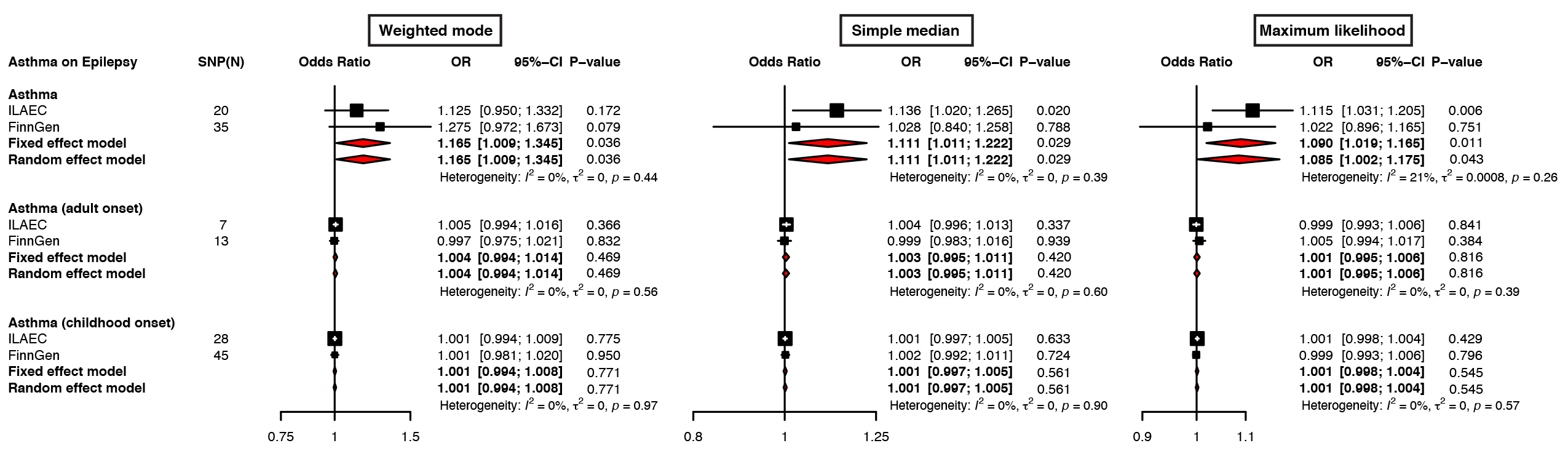
**

**Supplementary Figure S1.** Forest plots of meta-analysis on ILAEC and FinnGen epilepsy GWAS datasets show the causal effects of asthma on epilepsy using weighted mode, simple median, and maximum likelihood approaches. OR, odds ratio; CI, confidence interval; ILAEC, International League Against Epilepsy Consortium.
